# Active Indoor Soundscape Design: A Case Study of Ceramic Passive Amplifiers

**DOI:** 10.3390/ijerph191811251

**Published:** 2022-09-07

**Authors:** Feng Li, Jiali Xiang, Tao Li, Danni Shen, Tian Li

**Affiliations:** School of Art and Design, Zhejiang Sci-Tech University, Hangzhou 310018, China

**Keywords:** indoor soundscape, emotional perception, ceramic passive amplifier

## Abstract

Indoor soundscape research has developed rapidly in recent years, with the aim of improving the single indoor noise reduction method and people’s acoustic comfort. However, practical solutions to promote the generation of positive indoor soundscapes are still insufficient. The purpose of this study was to explore the improvement effect of ceramic passive amplifiers on the indoor soundscape and the relationship between the improvement effect and different amplifier shapes. Objective sound pressure level (SPL) values and subjective soundscape perception were measured for 10 ceramic passive amplifiers based on the soundscape, mainly using a comparative method. Ten sample amplifiers were compared with the acoustic data of the original open-plan studio environment, and then with an electronic sound amplifier. The results show that ceramic passive amplifiers can improve the quality of the indoor soundscape by creating sound scenes with appropriate loudness. Regarding non-acoustic aspects, the shape and materials of ceramic passive amplifiers play a positive role in emotional guidance.

## 1. Introduction

People’s health, behavioral performance, and well-being are strongly influenced by the indoor environment [1]. In the indoor environmental quality field, one of the most important factors is the acoustic environment, as a comfortable acoustic environment could influence physical and mental health directly [2]. Therefore, sounds and the indoor environment need to adapt to each other. Although many studies have proposed some methods of noise reduction for improving indoor acoustics, noise reduction still cannot satisfy people’s requirements for an acoustic environment. It is impossible to completely block noise and pure noise shielding sufficiently when dealing with the complex and changeable indoor environment. To improve the indoor acoustic environment (i.e., make it more flexible and effective), researchers have conducted many studies on the indoor soundscape by referring to the outdoor soundscape [3]. One approach is the suggestion that sounds should be considered as a resource rather than a waste [4]. However, existing research on the indoor soundscape is still focused on analyzing noise interference that causes people’s perception of “annoyance” and on developing noise reduction methods, and the scientific relationship between soundscape construction and people’s positive perception has not been concluded [5]. In addition, the mechanism of non-acoustic factors affecting indoor soundscapes is not clear.

Based on current developments in indoor soundscape research, there is still a need for more solutions that can stimulate positive perceptions. Improving indoor soundscapes can be shifted in the direction of coordinating various indoor sound sources and guiding people toward positive emotional reactions. A viable solution to create harmony in indoor soundscapes may be to use passive amplifiers. Firstly, by using passive amplifiers indoors to broadcast sounds that can lead to positive emotions and mask the sounds of other people, people would not worry about affecting others and could maintain normal communication [6]. Secondly, because passive amplifiers do not consume any energy, the indoor environment’s energy loss would not be increased, which meets the requirements of sustainable indoor soundscape development. In addition, the material and shape of the passive amplifier itself can be further optimized to increase the beauty of the indoor environment and strengthen the positive role of indoor non-acoustic factors. In this study, we utilize ceramic as a passive amplifier material, as it has good acoustic characteristics and a long history in musical instruments. Different styles of ceramic passive amplifiers have been designed to study the best shape for improving the indoor soundscape. Around this solution, this study raises the following questions: Could the indoor soundscape be improved when the ceramic passive amplifiers are added to the indoor space? Does the shape of the ceramic passive amplifier affect the improvement in the indoor soundscape?

## 2. Theoretical Background

### 2.1. Existing Soundscape Methods

A soundscape is defined as “the acoustic environment perceived or experienced or understood by one or more people in the context” [7]. A soundscape method provides a new direction for environmental acoustics. Specifically, traditional noise control research focuses on two aspects (i.e., noise reduction and sound isolation) [8], but it cannot satisfy people’s acoustic comfort directly. When people’s perception of sound is valued, acoustic comfort can truly be improved. Therefore, the soundscape method breaks the limitations of traditional noise control and tries to understand how people perceive sound from a perceptual perspective [9]. In other words, in the soundscape approach, the observed human perception is the main factor and objective acoustic measurement is the auxiliary factor. Based on the both factors’ observations, a flexible soundscape framework could be constructed to adjust the acoustic environment from a more comprehensive perspective. The soundscape framework also connects the context of the environment to conduct an overall analysis of the acoustic environment [10] so as to improve it over time and ultimately promote people’s health and well-being.

The goal of the soundscape approach is to develop and design sound as a common resource [11]. Indoor and outdoor soundscapes both belong to soundscape research, and their goal is same. Therefore, indoor soundscapes can learn from outdoor soundscapes, and appropriate sound resources can be selected for indoor spaces. Appropriate sound resources should take into account people’s perceptual preferences. Soundscape elements can be divided into three categories (i.e., natural, artificial, and mechanical sound). Artificial sounds are those created by human-related activities, such as talking, laughter and singing. Mechanical sounds are sounds produced by machines in human society, such as the sound produced by traffic vehicles and building construction. In general, people prefer artificial sounds to mechanical ones [12]. Natural sound has proven that it is the most favored soundscape element in many outdoor soundscape studies. Urban soundscapes are often designed and planned with natural soundscape elements, such as birdsong and water sounds [13]. People’s positive emotions can be activated by a natural soundscape to ward off feelings of fatigue and other negative emotions. This is known as biophilia. Specific sounds can bring people certain psychological energy and help refresh attention [14]. Therefore, when creating a positive indoor soundscape, appropriate natural sounds should be added to people’s positive sound perception to increase the beneficial impacts.

A positive indoor soundscape can cover up background noise, and people’s perceptions can successfully transition from a state of “noise annoyance” to “soundscape pleasure”. The sound environment of indoor space is complex and changeable, and it is impossible to keep quiet for a long time. As a too-quiet acoustic environment can also increase feelings of loneliness, the indoor environment requires sound resources with a certain loudness [15]. In addition, the degree of reverberation of the sound field in an indoor environment is also an important factor affecting people’s perception of sound loudness. When choosing a loudness sound, different values of the soundscape can be considered. Referring to the five conservation values of urban soundscape (i.e., ecological, comfort, emotional, identification, and practical value) [16], sound elements can be selected for an indoor soundscape with one or more values, which not only actively reconciles the indoor acoustic environment, but also adds non-acoustic value to the indoor environment. The development of indoor soundscapes has promoted a paradigm shift with regard to solving indoor environmental noise [17]. A positive perception of the indoor soundscape can stimulate a positive emotional response and improve sound quality and auditory comfort.

To explore whether ceramic passive amplifiers can create appropriate sound resources to improve indoor soundscape, we propose the following hypothesis.

**Hypothesis** **1.** The application of a ceramic passive amplifier in indoor space can effectively improve the quality of an indoor soundscape.

### 2.2. Passive Amplifier

According to the auditory masking effect [18], passive amplifiers can provide favored sounds to mask indoor noise and improve the soundscape. Therefore, utilizing passive amplifiers is feasible to create a good indoor soundscape. Passive amplifiers are also known as physical amplifiers. Specifically, their functions are based on the physical amplification principle with no power. The amplification principle of passive amplifiers is based on the characteristics of the nonlinear propagation of sound waves in the amplifier space [19], with sound waves guided to achieve the effect of diffusion of sound. A resonance cavity and a sound hole are the main components of a passive amplifier. When sound enters the enclosed resonating chamber, sound waves can only travel in the direction of the chamber, and waves in other directions are restricted. Since the energy of sound is fixed, the energy of sound waves can only be concentrated in the direction of the resonating cavity. A region of high pressure with low amplitude will occur in the resonator cavity. As sound waves travel through the sound hole of the amplifier, the accumulated energy is released and the amplitude increases, amplifying the sound over a long distance. The sound amplification effect of a passive amplifier can be enhanced by increasing the length of the resonating cavity and the area of the aperture. The design of the resonating cavity and sound hole is largely determined by the shape of the passive amplifier, for example, the common acoustic horn-shaped amplifier [20].

In recent years, research on passive amplifiers has been increasingly focused on material development and modeling innovation. In terms of materials, passive amplifiers mostly use hard materials, such as plastic, metal, etc. Because of the phenomenon of sound absorption in the sound wave transmission process and hard materials, the absorption rate is generally low. Generally, the surface of hard materials is smooth and flat, so the reflection and refractive indices of some acoustic waves can be appropriately increased to effectively expand the propagation range of acoustic waves. More recently, new materials such as silicone have been used to make passive speakers for smartphones. In terms of shape, trumpet-shaped amplifiers are typical for passive amplifiers, such as horn instruments [21] and tubular amplifiers [22]. With the evolution of design, the shape of passive amplifiers has fallen more in line with modern aesthetics (i.e., simple and ample), and are derived from a variety of shapes. As the use of smartphones has become a daily habit for people in modern life, the selection of an applicable object for a passive amplifier has focused on mobile phone products. As a result, passive amplifiers are becoming smaller in size in favor of a portable structure [23]. Passive amplifiers consume no energy and can play natural soundscapes, suitable for the “ecological value” of soundscapes. Passive amplifiers play sounds that people prefer, such as “natural sound”, which increases the “comfort value” of indoor acoustic. In terms of visual modeling, a passive amplifier with a beautiful shape can improve the “identification value” of an indoor soundscape and connect people’s emotions to the soundscape. Interior decoration can also include passive amplifiers, further improving the “practical value” of passive amplifiers.

In other words, different shapes of passive amplifiers have different sound amplification effects that affect the ability to mask indoor noise. Therefore, we propose the following hypothesis:

**Hypothesis** **1.1.** The acoustic amplification effect of a ceramic passive amplifier can affect the quality of an indoor soundscape.

### 2.3. Ceramic as Acoustic Material

Ceramic is chosen for the passive amplifiers in this study mainly because of its unique material characteristics and acoustic properties. Ceramic has special chemical properties, including high density and high hardness, which make it a hard kind of material. The surface of ceramic is usually covered with a glaze, so that it is smooth and flat. A warm and lustrous ceramic surface is visually appealing, giving it ornamental value. The unique acoustic properties of ceramic are also determined by these material characteristics. In the field of psychoacoustics, sound is evaluated in terms of loudness, pitch, rhythm, and timbre [24]. The loudness of sound transmitted by ceramic materials is suitable and the sound field is large. Using a ceramic passive amplifier, the pitch will not be too prominent, and a soft transition can be achieved. Timbre is the most notable aspect of the acoustic performance of ceramic materials. The sound transmitted by ceramic materials has excellent transparency, gentle sharpness, and low roughness [25]. The overall sound is crisp and distant, forming a unique sense of “sounding like a chime” [26], which is close to the natural sound. This kind of good sound experience will improve people’s evaluation of indoor soundscape quality to some extent as well.

The most common application of ceramic in the field of acoustics is musical instruments. Ceramic musical instruments take ceramic as the carrier of the musical performance and maximize its clear and pleasant acoustic characteristics. Most ceramic musical instruments were developed in China. Be it the earthen xun (a clay wind instrument with 11 holes) of primitive society, or a complete set of porcelain musical instruments in contemporary Jingdezhen (a city in Jiangxi Province), ceramic musical instruments have always occupied a place in history. In the earliest scientific classification of musical instruments of “eight sounds” in ancient China, ceramic instruments belong to the “indigenous” category [27]. In the West, many ancient ceramic instruments have been found in coastal Oaxaca, Mexico [28]. From the early ocarina to later ceramic flutes, various forms of instruments have been used to play a variety of music. Similarly, in the early 20th century, excavations in the ancient town of Numantia yielded pottery trumpets that best represent the Late Iron Age of the Iberian Peninsula, confirming the Celtic preference for ceramic instruments [29]. In modern times, utilizing hybrid ceramic composite as a coating for acoustic products is being considered [30]. Ceramic particles attached to the surface of the instrument reduce the acoustic response of interference, which can improve the acoustic quality and enhance the amplification effect. In this study, ceramics were used to make passive amplifiers in different shapes. The purpose of the study was to explore whether a passive amplifier could provide indoor sound that people want to hear and achieve positive soundscape subjective appraisals of “pleasantness” and “arousal” [31].

The beautiful shape and unique material acoustic characteristics not only enhance the sound amplification effect of passive amplifiers, but also attract people’s attention, guide people’s positive emotional perception, and finally achieve the purpose of improving the quality of indoor soundscape. Therefore, we propose the following hypothesis:

**Hypothesis** **1.2.** The shape of a ceramic passive amplifier can affect the evaluation of indoor soundscape quality.

### 2.4. Measurement Method

At present, indoor soundscape research methods mostly refer to outdoor soundscape methods, so research on indoor soundscapes includes data collection and reporting requirements as well [32]. The data collection mainly includes physical parameters, psychoacoustic metrics, and perceptual data.

Indicators including loudness, roughness, and sharpness are usually used in psychoacoustics [33], which plays a key role in auditory perception. The auditory perception of sound from the amplifier is mainly based on loudness, which is almost similar to the psychoacoustic index of the soundscape. Loudness refers to volume in daily life, and is a term used to judge the intensity of sound by the human ear. In many cases, the psychoacoustic indicators just describe the characteristics of sound, such as whether the sound is loud. Therefore, the actual acoustic data of the sound signal needs to be obtained by physical acoustic measurement. The most common technique in physical acoustics is sound level measurement, and the most commonly measured sound level parameter is the A-weighted sound pressure level [34]. Sound level values can be used to objectively evaluate the quality of the existing acoustic environment. Comparative analysis can be carried out to evaluate sound pressure values, focusing on differences in the soundscape before and after numerical changes. Objective data measurement of the acoustic performance of amplifiers also needs to use sound level values, which can be synchronized with soundscape measurement. When using passive amplifiers, it is also important to pay attention to the material, shape, and structure data, especially the length of the resonant cavity and the cross-sectional area of the sound hole.

Questionnaire surveys, sound walks, and narrative interviews are common methods for collecting perceptual data. The best way to accurately assess soundscape comfort in a particular location is to consult with local people [35]. Firstly, a questionnaire and interview can capture local sound preferences to extract the “pleasantness” or “annoyance” that they feel in the existing soundscape. Then, the questionnaire and interview content can be effectively summarized according to the perception degree of the soundscape descriptor, and the results can more accurately reflect the current quality of the soundscape. So far, the eight descriptors of the soundscape are: noise annoyance, pleasantness, quietness or tranquility, music alikeness, perceived affective quality, restrictiveness, soundscape quality, and appropriateness [36]. Additionally, non-acoustic factors (i.e., visual perception and the individual’s own conditions) are also recorded to enrich the soundscape assessment framework [37]. The final soundscape report needs to comprehensively analyze sound level data, subjective perception content, and non-acoustic factors to evaluate whether the created soundscape effectively improves the comfort level [38].

## 3. Ceramic Passive Amplifier Design

### 3.1. Design Concept

Designing a ceramic passive amplifier is an active exploration of the indoor soundscape, and also a modern expression of ceramic culture. This study attempts to improve the indoor soundscape through a passive amplifier. However, the passive amplifier cannot play the sound independently, and needs to use other sound players. In the living scene of an indoor environment, the smartphone is one of the most commonly used sound players. Therefore, smartphones are applicable objects for ceramic passive amplifiers which not only conform to the user’s habit of using mobile phones, but also adapt to the indoor scene. The sound amplification structure of the ceramic passive amplifier also needs to be designed according to the sound position and shape of the smartphone. At present, there are mobile phones of different sizes on the market, and the location of the sound hole differs. The average screen size of a smartphone increased from 4.86 inches in 2014 to 5.5 inches in 2018 [39]. The overall size of mobile phones is getting larger, so the size of the sound outlet of the passive amplifier must be adaptable. In this study, a 6.7-inch mobile phone was chosen as the standard to represent most mainstream mobile phones.

After confirming the applicable object, the modeling style of the ceramic passive amplifier had to be confirmed. The modeling style of amplifiers on the market can be roughly divided into traditional style and an imitation of the style of electronic speakers. Although there are some unique products, the overall style is still conservative. Therefore, in this design process, the ceramic passive amplifier had to break free from the conventional shape, and the style was in line with a modern and simple design. The sample modeling was more streamlined, using gradual transitions to avoid producing a structure that was too sharp. Ceramic shrinks in the firing process, changing the overall shape, and this uncontrollable factor could cause structural damage. Four categories of ceramic mobile phone amplifiers were designed based on the orientation of the resonant cavity structure: the first type was round tubular, and the direction of the sound hole was inclined to the front; the second type had a medium cavity, with the sound hole set at the bottom with multiple sound hole structures; the third type was characterized by a hollow two-tone structure with only one sound hole; and the fourth type had the sound hole above or on both sides.

### 3.2. Design Prototype

**Figure 1:**
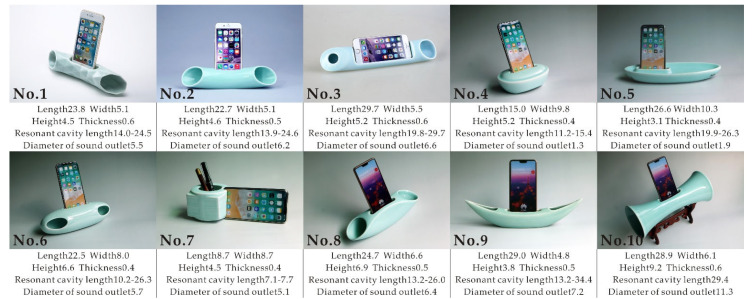
Details of experimental parameters of samples of ceramic mobile passive amplifiers (unit: cm).

In this study, 10 ceramic passive amplifiers with different shapes were designed for mobile phones, forming a series. Samples 1, 2, and 3 belonged to the first category of amplifiers, with a slender body. Samples 4 and 5 belonged to the second class of amplifiers, borrowing the features of ancient ingot and boats. Samples 6 and 7 belonged to the third category of amplifiers. The modeling of sample 7 referred to the ancient jade cong. Samples 8, 9, and 10 belonged to the fourth category of amplifiers. Samples 8 and 9 were modeled after a bridge and a canoe, respectively, and sample 10 was a trumpet shape. The overall style of the sample was essentially simple, personalized, interesting, and integrated into the daily life environment. The ceramic material was selected from Yaozhou Kiln, one of the six kilns from the Song Dynasty. The Yaozhou Kiln represents typical celadon in northern China. Its composition is thin and strong, its translucent glaze is smooth and subdued, faint green in color, and is considered very elegant. The whole series not only have the elegant feeling of ceramic ornaments, but also the sound effect of spreading melodious music. Thus, it was given the name “porcelain Ming”. The specific parameters of all samples are shown in Figure 1.

The resonant cavities of samples 4, 5, and 7 had a special shape. The shortest and longest distances of the outer frames of these samples were based on the measured resonant cavity length. The resonance cavities of the other samples were basically arc-shaped, and the measurements included the shortest and longest arc lengths. Except for sample 9, the sound holes of the other samples had an oval shape. Except for sample 7, all the other samples had symmetrical acoustic holes with the same area. Samples 4 and 5 contained more than two sound holes, and the size of these sound holes was similar. As calculating all of the sound holes’ areas was complex, we recorded the maximum diameter among all sound holes in each sample as the parameter of the sound hole area for each sample.

### 3.3. Experimental Design

In this study, 10 kinds of passive amplifiers were measured in four forward directions to provide objective data on their acoustic performance. The subjective soundscape perception of the amplifier samples was studied using a comparative method, and questionnaires and interviews were used to understand users’ perceptions. Finally, the objective data and subjective information were comprehensively analyzed to form the soundscape report and finally test hypothesis 1.

#### 3.3.1. Sound Environment Selection

**Figure 2:**
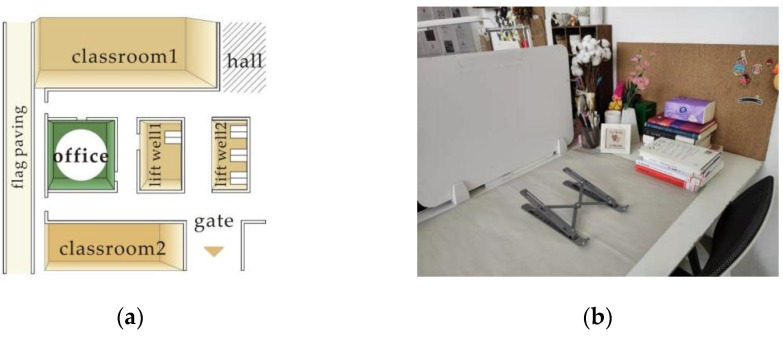
(**a**) Location of open-plan studio; (**b**) test environment in open-plan studio.

A virtual semi-anechoic room and a real open-plan studio were selected for the sound test environments. The semi-anechoic room was mainly intended to simulate a semi-free indoor acoustic field in order to avoid the interference of other sounds and obtain more accurate sound pressure level data of the 10 samples. Open-plan studios belong to the category of indoor soundscapes. The effect of ceramic passive amplifiers for mobile phones on the indoor soundscape can be seen with the use of an open-plan studio. The open-plan studio selected for this study was located on the middle-left side of the first floor of the building. A stone road passes alongside the window on the left side, and two elevator rooms are adjacent to the right side. The entrance and exit doors are in front of the elevator, so the background sound is complicated. The open-plan studio location and test environment are shown in Figure 2.

#### 3.3.2. Experimental Equipment

The equipment in this experiment was basically used to measure the sound levels and analyze the sound amplifier samples. The main equipment included a portable measurement and analysis system, a preamplifier, and a test microphone. A combination of a preamplifier and a test microphone was used to record the sound signals. In this study, the portable measurement and analysis system used a 1/3 octave frequency range, which is close to human perception, to analyze the sound-related levels (i.e., noise, vibration, etc.). A semi-anechoic room was used to reduce the interference of reflected sound, so the basic information of this room was also recorded. In the subjective soundscape perception test, an electronic speaker was used as one of the secondary devices in contrast to the sound perception of the passive amplifier. Details of the experimental equipment are shown in Table 1.

#### 3.3.3. Acoustic Measurement

In order to accurately evaluate the effect of ceramic passive amplifiers for mobile phones on the indoor soundscape based on objective data, the physical acoustic method was used to measure their sound pressure levels. Additionally, to test hypothesis 1.1, in the actual measurement process, in order to make the measured data better reflect people’s subjective feelings and correspond to the psychoacoustic loudness index, we mainly used the weighting method of sound pressure level A. The A-weighted sound pressure level method refers to the frequency weight, which is commonly used. Because the result measured by A-weighting is very close to the subjective feeling of the human ear, it can simulate people’s auditory perception of the sound made by the amplifier.

**Figure 3:**
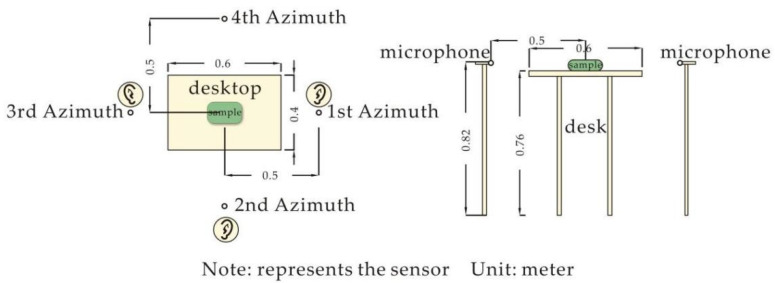
Layout diagram of instrument and equipment.

The 10 ceramic passive amplifier samples were placed on the middle of the table in the semi-anechoic room one by one. An NH-22A preamplifier and UC-57 test microphone were placed in four positive directions 0.5 m from the sample. The sequence arrangement of the four positive directions needs to simulate the listener’s auditory experience during actual use. As people to the front, left and right of the sound hole when the amplifier is in use, the right side of the sample sound hole was specified as the first azimuth; the second azimuth was determined as the front of the sample in a clockwise order; and the third and fourth azimuths were the left and rear, respectively. They were then connected to the SA-A1FTRTB4 portable measurement and analysis system (see Figure 3 for the layout of the equipment).

**Figure 4:**
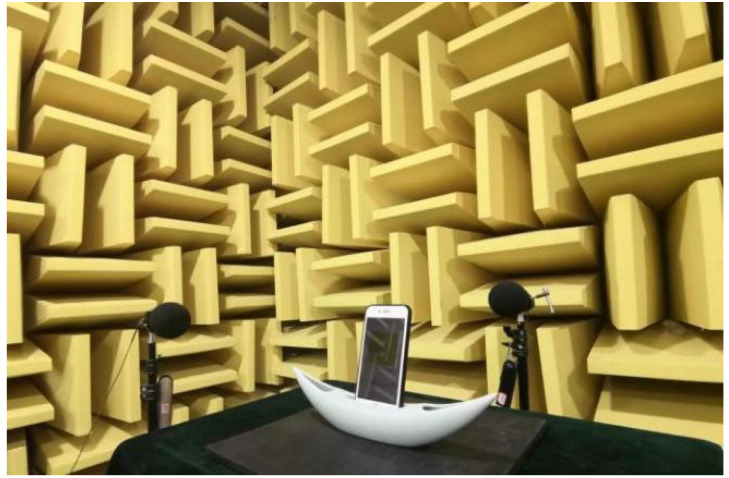
Actual shot of experimental scene (sample 9).

To begin the test, an iPhone 7 was placed in the middle of the table playing white noise (natural sound). The sound pressure of the four directions of the mobile phone at different frequencies was measured and compared with that of the subsequent sample. During the formal test, the iPhone 7 was inserted into the speaker groove of the sample (where the sound hole is), also playing white noise. The layout of the sample experiment is shown in Figure 4. Sound pressure level data of the four directions of the sample at different frequencies were collected. Each sample was measured a total of four times to obtain all A-weighted sound pressure level values.

Finally, the values of all A-weighted average sound pressure levels and A-weighted equivalent continuous sound pressure levels at different frequencies measured for four times were collated and tabulated. Both sound pressure levels are calculated directly by a portable measurement and analysis system for different purposes. The A-weighted average sound pressure level is the average value of the sound pressure levels of the sample at different frequencies. It is used to compare the sound amplification effect of the sample at low, medium and high frequencies and determine the influence of sample modeling on the sound amplification effect. The A-weighted equivalent continuous sound pressure level is a value that measures the average sound energy of an unsteady sound signal (usually a noise signal) over a period of time. It is used to compare the difference of the sound energy of mobile phones in different samples at the same time, and to measure whether the sample can mask indoor noise. Based on previous experiments, the typical value of background noise in an open-plan office is confirmed to be roughly between 52.0 and 58.0 dBA [40]. As the soundscapes of the selected open-plan studio and an open-plan office are similar, the typical noise values of the open-plan office were used in this study. The A-weighted equivalent continuous sound pressure level of the amplifier was compared with the typical value to confirm whether the samples could effectively mask the background noise of the open-plan studio.

#### 3.3.4. Soundscape Perception Experiment

The perception of the indoor soundscape where the ceramic passive amplifier was located was mainly obtained through questionnaires and interviews. The questionnaire first sought to understand the participants’ recognition degree of indoor sound source types under different sound conditions (Table 2), and then determine the noise masking effect of the samples. The change in emotional perception was the core content of the questionnaire. Referring to ISO guidelines for soundscapes, the questionnaire used a mood scale to describe differences in the soundscape. According to the soundscape descriptors, the semantic differences between six pairs of bipolar adjectives were set up on a mood scale and rated by a 5-point ordinal scale. The mood scale included two basic positive emotions (pleasant (high excitement) and calm (low excitement)) and two basic negative emotions (annoyance (high excitement) and dull (low excitement)). The other eight emotions were derived to enrich the emotion perception category (Table 3). The questionnaire ultimately required the participants to assess the overall soundscape quality of the open-plan studio (Table 4). Interviews and questionnaires were conducted simultaneously. Through guided interviews, the users’ overall evaluations of the sample were mined, including non-acoustic factors (visual perception, etc.), to perfect the evaluation results.

The time of the perception experiment was selected from 1 to 8 p.m. on weekdays. At this time, the ambient background sound of the open-plan studio was more complicated, and the shielding effect of the amplifier could be tested. The open-plan studio with no speakers and an electronic speaker were used as controls to compare the quality of the soundscape formed by the samples. In the test environment, smartphones were placed in 10 amplifier samples to play white noise and a popular song at the same volume (40%), while the position of the amplifier remained basically unchanged. The reason for choosing white noise was that according to soundscape theory, people prefer natural soundscapes, and the choice of a popular song was mainly because people are accustomed to creating indoor soundscapes in the form of songs. Since there was vegetation on the stone road on the left side of the open-plan studio, the white noise of birds and wind was in line with the characteristics of this environment. Each participant randomly selected the experimental sequence before the experiment, then experienced the sound environment. Participants experienced the sound environment of the open-plan studio, the sound environment while the white noise and popular song were playing from the electronic speaker, and all passive amplifier samples. The participants were asked to listen to 23 different sound environments. After each one, they were asked to fill out a corresponding questionnaire. Before listening to each pair of sounds, the participants rated the aesthetics of the passive amplifier samples to test hypothesis 1.2. Through the questionnaires and interviews, the perception of the soundscape with no amplifier, 10 passive amplifier samples, and an electronic amplifier in the same indoor environment could be compared to explore participants’ subjective perceptions of the samples.

## 4. Experimental Results

### 4.1. Evaluation of Acoustic Performance of Ceramic Passive Amplifiers for Mobile Phones

Table 5 shows the average sound pressure values of the 10 samples with low-frequency (below 500 Hz), medium-frequency (500–1600 Hz), and high-frequency (above 2000 Hz) sound signals. The average sound pressure values were the average values obtained by adding the A-weighted average sound pressure level values of the same frequency level under three frequency levels. First of all, with medium- and high-frequency sound signal input, the sound amplification effect of the 10 samples was obvious; with low-frequency sound signal input, the sound amplification effect was not good. In other words, all samples usually only enhanced sound radiation in a specific frequency range but could not cover a broad spectrum. Second, with regard to the structure of the ceramic passive amplifier, for the samples with a round tubular structure, given that the overall structure was basically the same, differences in the length of the cavity and the size of the sound hole affected the average sound pressure value of high-frequency sound signals. Moreover, the average sound pressure value of the sound signal in the middle-frequency band (near 1000 Hz) was significantly enhanced by the tubular structure, and the sound amplification effect of the sample was better. The samples with hollow cavity structure had weak sound conductivity, especially the shape with the sound hole at the bottom, and the overall sound amplification effect was poor. Sample 10 had a horn-like structure. The average sound pressure values measured at the horn-like sound holes were enhanced in the middle- and high-frequency bands (above 800 Hz), and the overall value was greater than that of the phone, which indicates that the resonator with horn-like structure could prevent the original sound from spreading in all directions. Moreover, by focusing on this in a limited resonator, it could spread the sound in the sound holes.

After analyzing the data, it was found that the data of the ceramic passive amplifiers with different shapes differed, and each sample had its own characteristics at different frequencies. The sound characteristics of the samples needed to be cross-compared, so a certain weight analysis method was used. As shown in Table 5, the scoring standard was determined as follows: in the same frequency band, if the average sound pressure value measured in the experiment after sound amplification was lower than that measured when the phone was not amplified, the score was 0; if the average sound pressure value after amplification was similar to that without amplification, the score was 1; and if the average sound pressure value after amplification was higher than that without amplification, the score was 2. When the individual hears the sound, it is mainly influenced by the first, second, and third azimuths of the sound amplifier, and the fourth azimuth has less influence. The direction of the sound hole of most amplifier samples was in the second azimuth, so the amplification effect of the second azimuth could best represent the performance of the sound amplifier. Therefore, the proportion of each azimuth’s score was specified as 50% for the second azimuth, 20% for the first and third azimuths, and 10% for the fourth azimuth. The weight analysis results are shown in Table 6, in which sample 2 has the highest score, and samples 1 and 10 also have high scores, which is consistent with the conclusion obtained from the analysis shown in Table 5. The score of sample 4 is the lowest, and there is a certain gap in sound amplification effect compared with other samples. This further proves that good acoustic performance requires a slender resonant cavity and a sound hole with moderate area.

Table 7 shows the average values of four A-weighted equivalent continuous sound pressure levels for all samples. These values represent the average sound energy in the four directions, simulating the human ear when listening to the amplified sound. In the first azimuth, the average values of the A-weighted equivalent continuous sound pressure levels of the mobile phone inserted in samples 4 (66.6 dB), 6 (67.9 dB), and 8 (66.9 dB) were lower than those of the mobile phone alone (68.2 dB), and the differences were 1.6, 0.3, and 1.3 dB, respectively. The sound amplification effect of sample 10 was the best, and the average value of the A-weighted equivalent continuous sound pressure level was up to 73.1 dB. In the second azimuth, samples 4 (69.5 dB), 7 (67.9 dB), 8 (67.6 dB), and 10 (67.8 dB) when the phone was inserted had lower average values of A-weighted equivalent continuous sound pressure level compared to the mobile phone alone (69.8 dB), and the differences were 0.3, 1.9, 2.2, and 2.0 dB, respectively. The maximum average value of A-weighted equivalent continuous sound pressure level was 73.7 dB for the phone inserted in sample 1, and the sound amplification effect was the best. The average values of the A-weighted equivalent continuous sound pressure levels of the inserted mobile phone in samples 4 (66.6 dB), 6 (66.5 dB), and 7 (65.8 dB) in the third azimuth were lower compared to the mobile phone alone (66.9 dB), and the differences were 0.3, 0.4, and 1.1 dB. The average value of the A-weighted equivalent continuous sound pressure level of the mobile phone inserted into sample 10 was 74.2 dB, which became the maximum value. In the fourth azimuth, all the samples had good sound amplification effect, and the average values of A-weighted equivalent continuous sound pressure levels were higher than those of the phone alone (62.1 dB). Among them, the sound amplification effect of sample 5 was most prominent, and the average value of A-weighted equivalent continuous sound pressure level was as high as 72.9 dB.

According to the data measured in the experiment, the A-weighted equivalent continuous sound pressure level values of all samples were above the maximum typical noise value of an open-plan office (58.0 dB) at the appropriate signal frequency, and had a masking function in objective conditions. Therefore, hypothesis 1.1 was partly supported. Moreover, the sound pressure of the sample in different directions was not too high, and was within the acceptable loudness range of the human ear.

### 4.2. Evaluation of Subjective Perception of Ceramic Passive Amplifiers

#### 4.2.1. Participants

Paid recruitment was adopted for this experiment, with a total of 30 questionnaire participants and 16 interviewees. In total, 12 male and 18 female college students participated in the experiment, ranging in age from 19 to 24 years, with a mean age of 22.40 years (SD = 1.27). All participants had normal vision and hearing perception and signed a consent form before the experiment began.

#### 4.2.2. Evaluation of Soundscape Improvement for Ceramic Passive Amplifiers

In order to explore the effect of ceramic passive amplifiers for mobile phones on improving the indoor soundscape, the experimental scores of the 10 samples were taken as a set of data, and the scores of no speakers and the electronic speaker were used as two other sets of data. One-way analysis of variance (ANOVA) was performed.

Table 8 and Table 9 show the differences in sound source perception. When white noise was played, all scores of the three groups for the sound environment were statistically significant (*p* < 0.01). When the sound playing was a popular song, the scores of the three groups for the sound environment still showed significant differences in “mechanical voice” and “artificial voice” (*p* < 0.01), while there was little difference in “natural sound” (*p* > 0.05) because popular songs do not contain “natural sound”.

In the post hoc significant difference (LSD) test, the score of “no speakers” was significantly lower than those of all samples and the electronic speaker (*p* < 0.05), and the scores of “natural sound” were significantly higher than those of all samples and the electronic speaker when white noise was played (*p* < 0.01). According to the score conversion of sound source recognition, the higher the score of noise (“mechanical voice”, “artificial voice”), the lower the score of “natural sound” and the better the noise shielding effect. Therefore, all samples and the electronic speaker achieved the effect of shielding the open-plan studio from ambient noise. For “natural sound” (when white noise is played), the score of the electronic speaker was significantly higher than that of sample 1 (M = 1.37, SD = 0.62; *p* < 0.01), which proves that sample 1 was better at transmitting “natural sound”. There was no significant difference in scores between the electronic speaker and all samples in terms of “mechanical voice” and “artificial voice” when playing a popular song (*p* > 0.05).

Table 10 and Table 11 show the results of assessing emotional perception. Whether white noise or a popular song was playing, all emotional scores of no speakers, the electronic speaker, and the 10 samples were statistically significant (*p* < 0.05). The post hoc LSD test showed that the “no speakers” scored significantly lower on six positive emotions (*p* < 0.05) and significantly higher on six negative emotions (*p* < 0.05) than all of the samples and the electronic speaker. The emotional score evaluation can be interpreted as follows: higher scores for six positive emotions or lower scores for six negative emotions indicate a higher comfort level of the soundscape. Therefore, the samples and the electronic speaker playing sound guided participants’ positive emotional perception.

Similarly, after the post hoc LSD test, there was no significant difference in the score for “pleasant” emotion between the electronic speaker and all samples when white noise was played (*p* < 0.05), indicating that the effect of the 10 samples in terms of guiding participants to perceive “pleasant” was similar to that of the electronic speaker. In terms of the scores for “calm”, “free”, and “familiar” emotions, the emotional score of seven samples was significantly higher than that of the electronic speaker (*p* < 0.05). In other words, the emotional guidance effect of the electronic speaker was worse than that of the passive amplifier in these positive emotions. The score of six positive emotions for sample 1 was significantly higher (*p* < 0.05) and the score of six negative emotions was significantly lower (*p* < 0.05) than that of the electronic speaker. The mood-improving effect of sample 1 went beyond the electronic speaker. There was no significant difference between the scores of positive “relaxed” and “lively” emotions for the other samples and the electronic speaker (*p* > 0.05) or the scores of the six negative emotions (*p* > 0.05). When a popular song was played, there was no significant difference between the electronic speaker and all samples in terms of emotional scores (*p* > 0.05).

**Figure 5:**
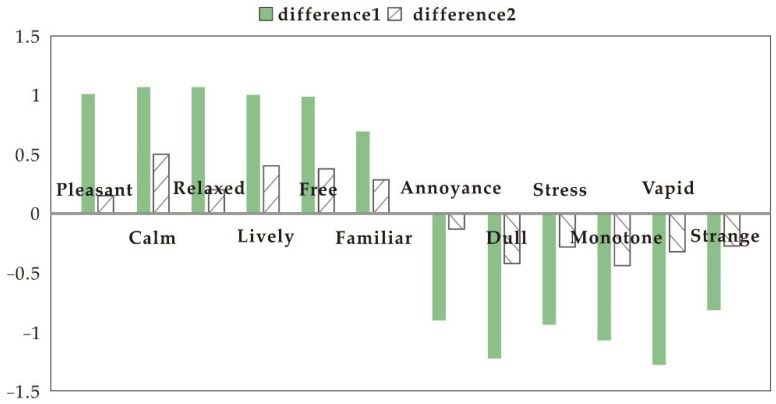
Differences in emotion perception (white noise). Difference 1: average difference in emotion perception between sample and no speakers; difference 2: average difference in emotion perception between sample and electronic speaker.

**Figure 6:**
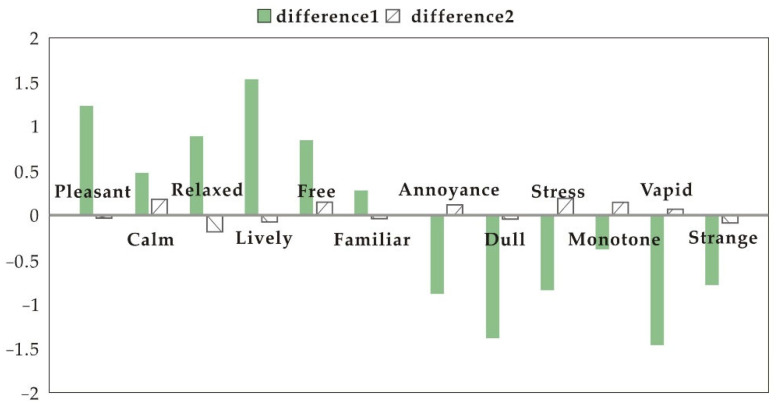
Differences in emotion perception (popular song). Difference 1: average difference in emotion perception between sample and no speakers; difference 2: average difference in emotion perception between sample and electronic speaker.

Figure 5 and Figure 6 show the calculations of differences in mood scores between the participants’ experience with no speakers, the samples, and the electronic speaker. When white noise was played, the overall difference between all samples and no speakers and the electronic speaker was significant. When playing a popular song, the overall difference between the samples and the no speakers was significant, and between the samples and the electronic speaker was not significant. When white noise was played, all samples were better at inducing positive perceptions than the electronic speaker. When playing a popular song, there was still a gap between all samples and the positive emotional guidance of the electronic speaker.

When white noise was played, the difference between “calm”, “relaxed”, “dull”, and “monotone” moods between the samples and no speakers was prominent. This suggests that soundscapes created by the samples were most helpful in restoring “calm” and “relaxed” moods, and prevented people from falling into “dull” and “monotone” moods. When playing a popular song, the difference between the samples and no speakers was significant in terms of “lively”, “dull”, and “monotone” moods. The results further confirm that all samples were most helpful in alleviating “dull” and “monotone” emotions.

Table 12 shows the differences in soundscape quality scores of the open-plan studios under the three conditions, and all scores are statistically significant (*p* < 0.01). In the post hoc LSD test, the soundscape quality scores of all samples and the electronic speaker were significantly higher than those of no speakers after playing white noise and a popular song (*p* < 0.01). The use of the samples and the electronic speaker successfully enhanced the soundscape quality of the open-plan studio. In the LSD test, there was no significant difference between the soundscape quality scores of the electronic speaker and all samples when playing a popular song (*p* > 0.05). When playing white noise, the soundscape quality scores of samples 1 and 2 were significantly higher than that of the electronic speaker (M = 4.00, SD = 0.53; M = 3.97, SD = 0.62; *p* < 0.01), indicating that samples 1 and 2 were better able to improve the indoor soundscape when playing “natural sound” than the electronic speaker.

#### 4.2.3. Evaluation of Soundscape Differences between Ceramic Passive Amplifiers

In order to compare the differences in soundscape evaluation among the 10 samples, the mood and soundscape quality data of all samples were integrated after playing white noise and a popular song.

Table 13 and Table 14 show the differences in mood scores of the 10 samples through descriptive analysis. The emotional scores of samples 1 and 2 were the most prominent among all the samples, followed by samples 9 and 10. The mood score of sample 4 was the worst among all samples, followed by samples 3 and 7. In the post hoc LSD test, the scores of four positive emotions of sample 1 were significantly higher (*p* < 0.05), and the scores of five negative emotions were significantly lower (*p* < 0.05) than those of sample 4. The “lively” and “free” scores of sample 1 were significantly higher (*p* < 0.05) and the “dull” and “monotone” scores were significantly lower (*p* < 0.05) than those of sample 5. The four positive emotions of sample 2 were significantly higher (*p* < 0.05) and the two negative emotions were significantly lower (*p* < 0.05) than those of sample 4. The “energetic” and “comfortable” scores of sample 2 were significantly higher than those of sample 5 (*p* < 0.05). In other words, the effect of mood improvement for samples 1 and 2 was more obvious compared to samples 4 and 5.

Table 15 shows the differences in soundscape quality assessment among the 10 samples. Sample 1 had the highest score, followed by samples 2 and 10. Sample 4 had the lowest score, followed by samples 3 and 7. In the LSD test, the soundscape score of sample 1 was significantly higher than those of samples 3 to 9 (*p* < 0.05), and the score of sample 10 was significantly higher than those of samples 3 to 8 (*p* < 0.05). Samples 1 and 10 had the best ability to improve the quality of the indoor soundscape, which is consistent with the objective acoustic performance of the two samples.

The influence of non-acoustic factors of the 10 samples on soundscape perception can be summarized from the interview process. Based on the subjective perception of respondents, emotional recovery was mentioned more frequently. Twelve interviewees clearly stated that they experience a “relaxed” feeling and entered a relatively “calm” state when white noise and a popular song were played with the 10 samples, and other sounds could be ignored. Fourteen respondents thought the sound transmitted by the samples was more ethereal than that of the electronic speaker. Three interviewees mentioned that the sound of the passive amplifier attracted attention and they could not concentrate on their thoughts to some extent.

From the perspective of sound effect, the amplification effect of samples 1 and 10 was easier to perceive, and eight interviewees thought that the reverberation effect created by sample 6 was the most obvious. The sound amplification effect of samples 4, 5, and 7 was evaluated by the respondents as being tedious. With regard to appearance, all respondents indicated that they were affected by the ceramic material and shape of the samples when evaluating the soundscape quality. Then, ten respondents clearly expressed a desire to touch the sample. Table 16 shows a comparison of the appearance scores of the samples.

Among them, the appearance of sample 1 had the highest score. When experiencing sample 1, respondents reported a corresponding increase in soundscape quality, which partly supported hypothesis 1.2. The appearance scores of samples 2, 8, and 9 were relatively high, and the corresponding soundscape quality scores were also relatively high. The appearance score of sample 4 was not low, but due to its poor amplification effect, the corresponding soundscape quality evaluation was not significantly improved. Due to the low appearance score of sample 10, the corresponding soundscape quality evaluation did not significantly improve. This shows that the visual and auditory perception of the samples were mutually influential.

Combining the results of the objective acoustic experiment and the subjective perception experiment, the improvement effect of ceramic passive amplifiers for mobile phones on the indoor soundscape was verified. Therefore, hypothesis 1 was supported. The specific experimental results are as follows:

1. The sound amplification effect of 10 samples was more obvious at medium frequency (500–1600 Hz) and high frequency (above 2000 Hz), and there was little difference in the effect of the mobile phone alone at low frequency (below 500 Hz). Among the samples, the sound amplification effects of samples 1, 2, and 10 were the most notable.
2. The 10 samples could play natural sound or music at the appropriate frequency, which can achieve the effect of masking indoor noise.
3. The indoor soundscape created by the 10 samples could help people enter a more “pleasant” and “relaxed” state and improve the overall quality of the indoor soundscape. The samples were more effective at inducing positive emotions when playing natural sounds than when playing an electronic speaker. Among the samples, samples 1 and 2 had the most positive effect on mood. Samples 4 and 5 showed an insufficient effect on guiding people’s positive perceptions.
4. In terms of non-acoustic effects, the sound transmitted by the ceramic material had a more restorative effect on emotional perception compared to the electronic speakers. On the premise of ensuring good acoustic performance, the recognizable aesthetic appearance of passive amplifiers is also conducive to the formation of a positive indoor soundscape.

## 5. Discussion

In this study, the experimental expectations and results were similar, and ceramic passive amplifiers were found to play an active role in the indoor soundscape. First of all, the sound amplification effect of the ceramic passive amplifiers in specific frequency bands was good, and the created sound atmosphere caused a change in the indoor soundscape that was positive as well. In fact, indoor ambient sound with ceramic passive amplifiers will increase in loudness. Although the added sound would not be classified as noise, it can be a positive sound source. The ceramic passive amplifier fully utilizes the characteristics of ceramic materials to produce a melodious and light sound, and can cover the noisy indoor acoustic environment for enhancing the indoor sound field. We found that the noise did not decrease. The potential reasons may be that the indoor noise had not been reduced, but the user’s attention was shifted to the sound played by the ceramic passive amplifier, and the user’s emotional perception of the soundscape tended to lean towards comfort. A noisy indoor acoustic environment with the addition of ceramic passive speakers creates a soundscape that is perceived as calm, pleasant, and relaxed. Among the 10 amplifier samples designed in this study, samples 1 and 2 had a long resonant cavity and a moderate sound hole area, which provided a good sound amplification effect and had the most obvious effect on noise shielding. However, the soundscape improvement effect was not ideal because the resonance cavity of samples 4 and 5 was short and the sound hole was located at the bottom.

Secondly, in terms of visual and tactile perception, ceramic materials not only decorate the interior space, but also enhance the sense of mild and calm, further realizing the research goal of creating a positive indoor soundscape. When evaluating the overall quality of the interior soundscape, the ceramic texture and aesthetic aspect promote the soundscape grade. The 10 ceramic passive amplifiers with different shapes follow modern design principles, break away from the traditional amplifier shape, and own simple and personalized style characteristics. The gloss and transparency of the ceramic adds visual comfort, and the flexible texture of the material contributes somewhat to the calm and relaxed soundscape atmosphere, which helps to restore the user’s attention. Samples 1 and 2 were found to have the best objective acoustic performance and subjective perceptual assessment in this study because they had good sound amplification effects and beautiful modeling performance. Although sample 10 had the best sound amplification effect, its shape was not well recognized, leading to a decline in its improvement of the indoor soundscape.

Improving indoor soundscape with ceramic passive amplifiers has achieved preliminary results, further proving that improving acoustic comfort is not only directly related to the number of sound sources, but also highly related to the quality of sound sources and environmental adaptability [41]. In this experiment, the complexity of the indoor soundscape did not decrease, but the natural soundscape was increased by the amplifier that harmonized the overall sound. A ceramic passive amplifier can bring a pleasant sound source into an interior area, and play a positive role in sound aspect and improving the quality of the soundscape. Similarly, introducing the sound of water and fountains in city parks [13] appropriately enhances the acoustic comfort of the parks. The conclusion that the acoustic comfort of indoor spaces cannot be improved by using noise reduction methods has also been found in other studies. We meet complex indoor acoustic environments in daily life, so information analysis and emotion-guided auditory perception need to be considered when people are experiencing such an environment. When evaluating the quality of the indoor soundscape added by the ceramic passive loudspeaker, we observed that the sound pressure level was improved in the objective aspect, and the users’ negative emotions were reduced in the subjective aspect. At the same time, the acoustic comfort was improved.

The use of ceramic passive amplifiers in indoor soundscapes draws upon the auditory masking effect in urban soundscape planning. According to the characteristics of the indoor environment, the sounds of wind and birds are played, and indoor noises are properly blocked out, guiding people to pay attention to positive natural sounds. The addition of natural sounds should be adapted to the indoor acoustic environment, rather than forcing in disagreeable sounds [42]. Therefore, people will not be particularly resistant to the addition to the soundscape, and will easily accept the positive emotional guidance. In addition, the soundscape should be consistent with the visual message conveyed by the amplifier. The use of ceramic materials in passive amplifiers makes people feel calmer, which is consistent with the goal of creating a comfortable soundscape in the indoor environment. Many reports have examined the role of vision in auditory judgment, as people’s appreciation of the sound environment is positively influenced by the visual aspects of plants and natural elements [43].

## 6. Conclusions

This study introduces the active sound source of ceramic passive amplifiers to improve the comfort of indoor soundscapes and provide a solution for the creation of a positive indoor soundscape. In this study, 10 kinds of ceramic passive amplifier shapes for mobile phones were designed to create a loudness soundscape for improving the indoor acoustic environment. The acoustic performance of the ceramic passive amplifiers proved to be good by measuring the sound pressure levels of the samples. Then, a questionnaire survey and guided interviews were conducted with study participants. The soundscapes created by the samples were compared with those created by no speakers and an electronic speaker, which proved that the addition of a ceramic passive amplifier to the indoor soundscape played a positive role in guiding people’s emotions and effectively and improved the quality of the indoor soundscape. This study provides practical tips for improving indoor soundscapes. Firstly, under the appropriate sound frequency, ceramic passive amplifiers have an obvious sound amplification effect and enhance the reverberation effect of the indoor sound field, better shielding the indoor noise. Secondly, the sound created by ceramic materials is more ethereal in timbre, which enhances the calming effect of the visual psychological level and increases the “identification value” of the indoor soundscape. The unique shape of ceramic passive amplifiers also improves the feasibility of creating a positive indoor soundscape and provides a reference on how to improve the quality of the indoor soundscape by using non-acoustic factors. Finally, the ceramic passive amplifiers were found to successfully improve the soundscape quality of an open-plan studio by playing different sounds (white noise and popular song), which also provides a reference on how to create sound “resources” that guide people’s positive perceptions.

This study demonstrates that the acoustic performance of ceramic passive amplifiers makes them a feasible suggestion for creating a positive indoor soundscape. However, this conclusion needs further validation. The indoor scene used in this experiment was relatively singular, and there are many variables of sound sources. It is unknown whether the use of an amplifier in other indoor scenes is feasible. Some sample designs in this study showed a poor sound amplification effect during sound level measurement. The resonant cavity of the amplifier should have enough space, and the sound hole should not be located at the bottom or the top to meet the listeners’ using habits. The shape of the amplifier could be further improved, and more possibilities for ceramic materials to shape the structure of the amplifier should be explored on the premise of good sound reinforcement performance. The introduction of passive amplifiers in indoor acoustics should also consider sound effects, and the advantage of electronic speakers for sound effects cannot be ignored. Improving the sound effect of passive amplifiers could fully utilize the sound characteristics of materials and create special timbre and reverberation in the sound field. Utilizing ceramics in passive amplifiers for mobile phones also extends in the direction of modern ceramic applications, and appropriately introduces the concept of using materials that evoke a subdued visual psychological perception to improve the indoor soundscape. In further research, we plan to create passive amplifiers with other milder materials to promote the positive development of the non-acoustic aspect in indoor soundscapes.

## Figures and Tables

**Table 1:** Experimental equipment parameters.

Name of Instrument	Model	Factory Number	Number	Manufacturer	Equipment Number
Portable measurement and analysis system	SA-A1FTRTB4	01170458	1	Brüel & Kjær	STS-YQ/N0048
Preamplifier + test microphone	UC-57/NH-22A	00573 + 80306	4	/	STS-YQ/N0050
		00573 + 80307			STS-YQ/N0051
		00573 + 80308			STS-YQ/N0052
		00573 + 80309			STS-YQ/N0053
Semi-anechoic room	JYT-0922B	20170922	1	/	STS-YQ/L0003
Temperature and humidity meter	/	/	1	/	STS-YQ/C0037
Mao King FM/Bluetooth portable speaker	MW-2A	180206943716	1	Shenzhen Cloud Motion Creative Technology Co., Ltd. (Shenzhen, China)	/

**Table 2:** Sound source identification in open-plan studio environment.

Sound Source Type	Mechanical Voice		Artificial Voice	Natural Sound	
Recognition degree	Not at all	A little	Moderately	A lot	Dominates completely
Score conversion	5	4	3	2	1

**Table 3:** Mood scale.

Emotional Category	Pleasant	Calm	Relaxed	Lively	Free	Familiar
	Annoyance	Dull	Stress	Monotone	Vapid	Strange
Perceived level	Very much agree	Agree	General	Do not agree		Strongly disagree
Score conversion	5	4	3	2		1

**Table 4:** Overall assessment of open-plan studio’s sound environment.

Soundscape Quality	Very Satisfied	Relatively Satisfied	General	Relatively Dissatisfied	Very Dissatisfied
Score conversion	5	4	3	2	1

**Table 5:** Average sound pressure values of 10 samples in four azimuths at different frequencies (dbSPL).

Sample	Frequency Range	1st Azimuth Mean	2nd Azimuth Mean	3rd Azimuth Mean	4th Azimuth Mean
1	Low frequency	22.1	22.7	18.7	18.9
	Medium frequency	50.3	55.7	50.5	53.1
	High frequency	54.2	58.8	55.4	51.0
2	Low frequency	26.3	26.3	20.8	20.7
	Medium frequency	50.2	55.6	50.5	53.1
	High frequency	53.7	58.1	55.7	52.5
3	Low frequency	24.3	25.5	25.8	25.1
	Medium frequency	50.3	53.2	51.1	49.5
	High frequency	54.4	55.1	51.1	49.2
4	Low frequency	24.8	25.3	23.1	23.4
	Medium frequency	49.3	46.4	51.2	47.9
	High frequency	51.7	55.9	51.6	51.6
5	Low frequency	22.1	22.7	23.4	22.6
	Medium frequency	48.1	48.7	48.7	49.3
	High frequency	56.1	56.3	56.0	56.9
6	Low frequency	19.9	20.9	20.4	20.2
	Medium frequency	49.3	54.2	49.9	51.1
	High frequency	53.3	57.9	51.1	50.0
7	Low frequency	24.2	24.1	24.6	24.5
	Medium frequency	51.4	50.0	51.4	49.0
	High frequency	52.6	52.6	50.4	52.9
8	Low frequency	22.9	23.0	24.1	24.5
	Medium frequency	48.7	52.5	49.3	52.3
	High frequency	52.7	52.5	54.5	51.4
9	Low frequency	22.9	22.6	24.4	24.3
	Medium frequency	52.9	51.6	53.0	52.8
	High frequency	53.9	53.5	53.3	53.5
10	Low frequency	24.8	25.7	22.6	22.5
	Medium frequency	54.6	54.3	54.3	54.7
	High frequency	59.7	52.4	60.9	49.4
Mobile phone	Low frequency	26.4	25.8	26.6	26.2
	Medium frequency	48.4	50.3	47.5	42.5
	High frequency	53.0	55.6	52.0	48.6

**Table 6:** Weight analysis data of 10 samples.

Sample	1	2	3	4	5	6	7	8	9	10
Score	2.6	3.1	1.4	1.2	1.5	1.9	1.5	1.4	1.9	2.9

**Table 7:** Average values of A-weighted equivalent continuous sound pressure level for 4 azimuths of 10 samples (dbSPL).

Sample Type	Sample	1st Azimuth Mean	2nd Azimuth Mean	3rd Azimuth Mean	4th Azimuth Mean
First type	1	68.8	73.7	69.7	68.9
	2	68.7	73.1	69.8	68.6
	3	68.2	70.6	67.3	64.6
Second type	4	66.6	69.5	66.6	65.8
	5	70.1	70.4	70.6	72.9
Third type	6	67.9	72.5	66.5	66.4
	7	70.0	67.9	65.8	65.9
Fourth type	8	66.9	67.6	68.6	67.5
	9	69.9	69.8	69.7	70.2
	10	73.1	67.8	74.2	67.5
/	Mobile phone	68.2	69.8	66.9	62.1

**Table 8:** Differences in sound source identification between no speakers, samples, and electronic speaker (white noise).

Sound Source Type	No Speakers		Electronic Speaker		Samples of 1 to 10		F *	df1;df2	*p* *
	Mean	SD *	Mean	SD *	Mean	SD *			
Mechanical voice	2.77	1.04	3.63	0.85	3.36	0.97	2.475	11;348	0.005
Artificial voice	2.33	0.80	2.83	0.79	3.24	0.95	3.756	11;348	0.000
Natural sound	4.40	0.97	2.40	1.19	2.13	1.23	10.651	11;348	0.000

* SD, standard deviation; F, Brown–Forsythe ANOVA; fd, freedom degree; *p*, probability value.

**Table 9:** Differences in sound source identification between no speakers, samples, and electronic speaker (popular song).

Sound Source Type	No Speakers		Electronic Speaker		Samples of 1 to 10		F *	df1;df2	*p* *
	Mean	SD *	Mean	SD *	Mean	SD *			
Mechanical voice	2.77	1.04	3.70	0.88	3.83	0.90	3.824	11;348	0.000
Artificial voice	2.33	0.80	3.20	0.89	3.28	0.91	3.417	11;348	0.000
Natural sound	4.40	0.97	4.53	0.86	4.45	1.10	0.167	11;348	0.999

* SD, standard deviation; F, Brown–Forsythe ANOVA; fd, freedom degree; *p*, probability value.

**Table 10:** Differences between 12 emotional perceptions under different sound conditions (white noise).

Emotional Category	No Speakers		Electronic Speaker		Samples of 1 to 10		F *	df1;df2	*p* *
	Mean	SD *	Mean	SD *	Mean	SD *			
Pleasant	2.87	0.63	3.73	0.83	3.88	0.74	5.500	11;348	0.000
Calm	2.80	1.03	3.37	0.89	3.87	0.83	5.390	11;348	0.000
Relaxed	3.00	0.91	3.87	0.82	4.07	0.74	6.115	11;348	0.000
Lively	2.50	1.04	3.10	0.92	3.50	0.87	4.946	11;348	0.000
Free	2.97	0.89	3.57	0.77	3.95	0.69	6.695	11;348	0.000
Familiar	3.03	1.07	3.43	1.01	3.72	0.81	3.290	11;348	0.000
Annoyance	3.00	0.83	2.23	0.86	2.10	0.77	4.565	11;348	0.000
Dull	3.47	0.97	2.67	0.84	2.25	0.87	6.653	11;348	0.000
Stress	2.73	0.98	2.07	0.94	1.79	0.75	4.750	11;348	0.000
Monotone	3.63	1.10	3.00	0.95	2.56	0.91	4.347	11;348	0.000
Vapid	3.63	1.10	2.67	0.66	2.35	0.84	7.353	11;348	0.000
Strange	2.97	1.13	2.43	0.86	2.16	0.81	3.066	11;348	0.001

* SD, standard deviation; F, Brown–Forsythe ANOVA; fd, freedom degree; *p*, probability value.

**Table 11:** Differences between 12 emotional perceptions under different sound conditions (popular song).

Emotional Category	No Speakers		Electronic Speaker		Samples of 1 to 10		F *	df1;df2	*p* *
	Mean	SD *	Mean	SD *	Mean	SD*			
Pleasant	2.87	0.63	4.13	0.63	4.10	0.71	9.166	11;348	0.000
Calm	2.80	1.03	3.10	0.80	3.28	0.94	1.860	11;348	0.044
Relaxed	3.00	0.91	4.07	0.74	3.89	0.83	3.938	11;348	0.000
Lively	2.50	1.04	4.10	0.80	3.50	0.75	11.679	11;348	0.000
Free	2.97	0.89	3.60	0.86	3.82	0.80	3.635	11;348	0.000
Familiar	3.03	1.07	3.43	1.01	3.58	0.88	1.756	11;348	0.060
Annoyance	3.00	0.83	2.00	0.74	2.12	0.78	4.255	11;348	0.000
Dull	3.47	0.97	2.13	0.86	2.09	0.84	7.549	11;348	0.000
Stress	2.73	0.98	1.70	0.79	1.89	0.82	3.294	11;348	0.000
Monotone	3.63	1.10	2.10	0.89	2.25	0.84	6.930	11;348	0.000
Vapid	3.63	1.10	2.10	1.00	2.17	0.87	7.253	11;348	0.000
Strange	2.97	1.13	2.27	0.74	2.19	0.76	2.547	11;348	0.004

* SD, standard deviation; F, Brown–Forsythe ANOVA; fd, freedom degree; *p*, probability value.

**Table 12:** Comprehensive evaluation of soundscape quality.

Emotional Category	No Speakers		Electronic Speaker		Samples of 1 to 10		F *	df1;df2	*p* *
	Mean	SD *	Mean	SD *	Mean	SD *			
White noise	2.23	0.63	3.33	0.55	3.63	0.62	14.667	11;348	0.000
Popular song	2.23	0.63	3.73	0.58	4.03	0.62	15.809	11;348	0.000

* SD, standard deviation; F, Brown–Forsythe ANOVA; fd, freedom degree; *p*, probability value.

**Table 13:** Differences in positive emotion perception among samples.

Emotional Category	Pleasant		Calm		Relaxed		Lively		Free		Familiar	
	Mean	SD*	Mean	SD *	Mean	SD *	Mean	SD *	Mean	SD *	Mean	SD *
1	4.23	0.70	3.60	1.08	4.25	0.75	4.07	0.78	4.05	0.62	3.63	0.84
2	4.20	0.68	3.60	1.09	4.08	0.81	4.03	0.78	4.03	0.84	3.58	0.85
3	3.97	0.71	3.47	0.98	4.00	0.88	3.63	0.84	3.95	0.85	3.92	0.85
4	3.77	0.70	3.38	0.98	3.75	0.84	3.47	0.89	3.62	0.76	3.43	0.81
5	4.00	0.69	3.60	0.96	4.02	0.65	3.60	0.85	3.72	0.72	3.62	0.83
6	3.93	0.78	3.67	1.00	4.00	0.80	3.78	0.90	3.93	0.80	3.57	1.02
7	3.90	0.75	3.55	0.87	3.87	0.83	3.70	0.87	3.80	0.66	3.52	0.87
8	3.92	0.72	3.55	0.79	3.83	0.76	3.68	0.91	3.78	0.78	3.60	0.76
9	4.00	0.78	3.67	0.80	3.92	0.77	3.75	0.82	4.00	0.66	3.78	0.76
10	3.95	0.72	3.65	0.76	4.08	0.77	3.90	0.78	3.97	0.66	3.83	0.79

* SD, standard deviation; F, Brown–Forsythe ANOVA; fd, freedom degree; *p*, probability value.

**Table 14:** Differences in negative emotion perception among samples.

Emotional Category	Annoyance		Dull		Stress		Monotone		Vapid		Strange	
	Mean	SD *	Mean	SD *	Mean	SD *	Mean	SD *	Mean	SD *	Mean	SD *
1	1.80	0.68	1.88	0.72	1.73	0.78	2.20	0.84	1.92	0.77	2.07	0.88
2	2.03	0.76	1.97	0.80	1.77	0.89	2.28	0.80	2.15	0.92	2.13	0.85
3	2.17	0.91	2.22	0.94	1.90	0.84	2.45	0.93	2.37	0.90	2.22	0.78
4	2.25	0.80	2.53	0.93	2.07	0.80	2.60	0.89	2.43	0.87	2.20	0.76
5	2.10	0.75	2.15	0.86	1.80	0.82	2.47	1.00	2.32	0.97	2.18	0.87
6	2.30	0.74	2.22	0.99	1.98	0.81	2..37	0.97	2.25	0.91	2.23	0.83
7	2.12	0.74	2.28	0.87	1.78	0.76	2.47	0.87	2.43	0.85	2.27	0.80
8	2.08	0.70	2.15	0.71	1.73	0.73	2.40	0.85	2.30	0.74	2.15	0.63
9	2.05	0.70	2.13	0.85	1.77	0.70	2.40	0.87	2.25	0.77	2.18	0.75
10	2.20	0.86	2.13	0.77	1.87	0.72	2.42	0.85	2.20	0.84	2.10	0.71

* SD, standard deviation; F, Brown–Forsythe ANOVA; fd, freedom degree; *p*, probability value.

**Table 15:** Differences in soundscape quality assessment of 10 samples.

Sample	1	2	3	4	5	6	7	8	9	10
Mean	4.03	3.82	3.52	3.38	3.60	3.77	3.57	3.57	3.73	3.83
SD	0.64	0.65	0.70	0.72	0.62	0.72	0.72	0.59	0.58	0.64

**Table 16:** Average score of appearance evaluation of 10 samples (10-point system).

Sample	1	2	3	4	5	6	7	8	9	10
Mean	7.67	7.07	5.97	6.23	6.90	6.73	6.53	7.23	7.30	6.57
SD	1.52	1.57	1.43	1.83	1.35	0.91	1.47	1.07	1.37	1.43

## Data Availability

The data used to support the findings of this study are available from the corresponding author upon request.
